# Clinical and Geriatric Predictors of In-Hospital Mortality in Older Adults Admitted to Internal Medicine Wards: A Retrospective Observational Study

**DOI:** 10.3390/jcm14196726

**Published:** 2025-09-24

**Authors:** Carmine Siniscalchi, Pierpaolo Di Micco, Angela Guerra, Antonio Nouvenne, Nicoletta Cerundolo, Alberto Parise, Tiziana Meschi

**Affiliations:** 1 Internal Medicine Department, Parma University Hospital, 43120 Parma, Italy; csiniscalchi84@gmail.com (C.S.); angela.guerra@unipr.it (A.G.); antonio.nouvenne@unipr.it (A.N.); ncerundolo@ao.pr.it (N.C.); aparise@ao.pr.it (A.P.); tiziana.meschi@unipr.it (T.M.); 2 Internal Medicine, Ward Santa Maria delle Grazie, Pozzuoli Naples Hospital 2 Nord, 80078 Naples, Italy

**Keywords:** older adult, frailty, delirium, in-hospital mortality, sex differences

## Abstract

**Background**: Older adults who are hospitalized in internal medicine wards often present with a challenging interplay of multimorbidity and geriatric syndromes. The timely identification of clinical and geriatric predictors of in-hospital mortality is crucial for guiding individualized care pathways and ensure appropriate resource allocation. In this study, we investigate the prognostic impact of frailty, delirium—including its motor subtypes—and global comorbidity burden on in-hospital mortality in patients aged 70 years and older. **Methods**: We conducted a retrospective observational study including 556 consecutive patients aged ≥ 70 years who were admitted to the Internal Medicine Unit of the University Hospital of Parma from January 2019 to July 2019. Demographic, clinical, and geriatric data were collected within 48 h of admission, including Clinical Frailty Scale (CFS), Cumulative Illness Rating Scale (CIRS), and delirium diagnosis with the 4AT tool. Multivariate Cox and logistic regression analyses were performed, including sex-stratified models. **Results**: The median age was 85 years (IQR 80–89), 58% were female, and in-hospital mortality was 11% (n = 61). Non-survivors had higher rates of severe frailty (CFS ≥ 7: 39% vs. 16%, *p* < 0.001), prevalent delirium (20% vs. 4%, *p* < 0.001), hypokinetic delirium (20% vs. 5%, *p* < 0.001), liver disease (23% vs. 11%, *p* = 0.008), cancer (44% vs. 24%, *p* < 0.001), and dementia (43% vs. 29%, *p* = 0.026) and a higher CIRS severity index (≥3:55% vs. 31%, *p* < 0.001). In Cox regression, independent predictors of death were prevalent delirium (HR 4.66, 95% CI 2.42–8.96), CFS ≥ 7 (HR 2.26, 95% CI 1.32–3.87), CIRS-LIVER ≥ 2 (HR 2.05, 95% CI 1.18–3.56), and cancer (HR 1.83, 95% CI 1.07–3.14). Sex-stratified models showed that in males, prevalent delirium (HR 10.23) and cancer (HR 2.49) predicted mortality, whereas in females, hypokinetic delirium (HR 3.67) and CIRS-LIVER ≥ 2 (HR 2.75) were the strongest predictors. Logistic regression confirmed these associations and additionally identified anemia and CFS ≥ 7 in males and CIRS severity index ≥ 3 in females as significant risk factors. **Conclusions**: In elderly patients who are admitted to internal medicine wards, prevalent and hypokinetic delirium, severe frailty, and high comorbidity burden, particularly liver disease and cancer, are strong independent predictors of in-hospital mortality, with distinct sex-specific patterns. Early multidimensional geriatric assessment may improve risk stratification and guide targeted interventions.

## 1. Introduction

The progressive aging of the population is leading to a growing number of older adults being admitted to internal medicine wards, where multimorbidity, polypharmacy, and complex geriatric syndromes, most notably frailty and delirium, are highly prevalent. Such conditions require a patient-centered approach that transcends disease-focused models, as geriatric factors critically influence both prognosis and functional outcomes [1,2]. In-hospital mortality in this population reflects the combined effects of acute illnesses and reduced physiological reserve. Relying exclusively on demographic or diagnostic variables often fails to accurately predict prognostic risk in this vulnerable population [3]. The inclusion of frailty and cognitive status in prognostic models has been shown to markedly improve risk stratification [4,5]. Frailty, most frequently assessed through the Clinical Frailty Scale (CFS), is a robust predictor of mortality. Severe frailty significantly increases the risk of death, prolonged hospital stays, and subsequent institutionalization [3]. Delirium, an acute and often underrecognized neurocognitive syndrome, is consistently associated with adverse outcomes, including increased mortality, functional decline, and higher rates of rehospitalization [6,7]. Despite increasing awareness, multidimensional geriatric assessment is still underutilized in internal medicine, especially in acute care settings [8]. Moreover, there is limited evidence on the combined prognostic role of frailty, delirium—including its subtypes—and comorbidity burden (especially chronic liver disease and cancer) in real-world hospitalized populations. This study aims to fill that knowledge gap by exploring the clinical and geriatric predictors of in-hospital mortality in patients aged 70 years and older who are admitted to an internal medicine ward, with a specific focus on frailty, delirium (including prevalent and hypokinetic forms), comorbidity severity, and sex-specific prognostic differences.

## 2. Materials and Methods

This retrospective observational study was conducted at the Internal Medicine Unit of the University Hospital of Parma, Italy. All patients aged 70 years or older who were consecutively admitted from March 2019 to July 2019 were screened for eligibility. The study was approved by the local ethics committee and conducted in accordance with the principles outlined in the Declaration of Helsinki. Written informed consent was obtained from each participant or, when necessary, from a legal representative. Eligible participants were patients aged ≥ 70 years who were admitted for any acute medical condition. Exclusion criteria were admission from surgical wards or intensive care units and missing data on key clinical or geriatric variables. A total of 556 patients were included in the final analysis (231 males and 325 females). Within the first 48 h of admission, demographic information (age, sex), clinical history, number of chronic conditions, and daily medications were recorded. Comorbidity burden was assessed using the Cumulative Illness Rating Scale (CIRS), including the Comorbidity Score (CIRS-CS) and the Severity Index (CIRS-SI). The primary admission diagnosis was classified into predefined groups (pneumonia, cardiovascular, infectious, and others). Frailty was assessed using the Clinical Frailty Scale (CFS), with a score ≥ 7 indicating severe frailty [9]. Delirium was screened using the 4AT tool, a validated rapid diagnostic instrument for older hospitalized adults [10]. Delirium subtypes were classified as hyperkinetic, hypokinetic, or mixed according to motor and arousal features [11]. All assessments were performed by trained physicians using standardized case report forms. The early application of validated geriatric scales is recommended for risk stratification in acutely hospitalized older adults [12]. The primary outcome was all-cause in-hospital mortality, defined as death occurring during the index hospitalization. Mortality data were obtained from electronic health records and confirmed through discharge summaries.

Continuous variables were reported as median and interquartile range (IQR), while categorical variables were expressed as percentages and sometimes in absolute numbers. The Mann–Whitney U test was used for comparisons of continuous variables, chi-square, Yates’ chi-square, or Fisher’s exact test for categorical variables. Multivariable Cox proportional hazards regression and logistic regression models were then applied to identify independent predictors, adjusting for potential confounders. Stepwise methods were used to optimize variable selection. Additional analyses stratified by sex were conducted to explore gender-specific prognostic patterns. The tree classification of the study population for in-hospital mortality was applied (CHAID method). A *p* value < 0.05 was considered statistically significant. Inclusion criteria were age ≥ 70 years, consecutive medical admissions, and availability of geriatric assessment within 48 h. Exclusion criteria were missing key geriatric variables. For Cox models, proportional hazards assumptions were tested. All statistical analyses were conducted using SPSS version 28.0 (IBM Corp., Armonk, NY, USA).

## 3. Results

A total of 556 patients aged ≥ 70 years were included in the study, with a median age of 85 years (IQR 80–89) and with 58% being female. The overall in-hospital mortality was 11% (n = 61). Table 1 summarizes the baseline characteristics of the study population according to sex. Males were significantly younger than females (median 83 vs. 86 years, *p* = 0.001) but exhibited a higher burden of several comorbidity domains, including vascular, liver, kidney, and urologic diseases, as well as higher overall CIRS Comorbidity Scores and Severity Index values (*p* = 0.001 and *p* < 0.001, respectively). Chronic obstructive pulmonary disease (COPD), diabetes, chronic kidney disease (CKD), peripheral artery disease, liver disease, and cancer were more frequent among men, whereas osteoporosis, arthrosis, and dementia were more prevalent in women. No significant sex differences were observed in the distribution of primary admission diagnoses, hospital length of stay, or mortality rates. Incident delirium occurred more frequently in females (21% vs. 14%, *p* = 0.032).

When stratified by survival status (Table 2), non-survivors had significantly higher CFS scores (median 6 vs. 5, *p* < 0.001), a greater prevalence of severe frailty (CFS ≥ 7: 39% vs. 16%, *p* < 0.001), and higher CIRS vascular, liver, psychiatric, Comorbidity Score, and Severity Index values. Chronic liver disease (23% vs. 11%, *p* = 0.008), cancer (44% vs. 24%, *p* < 0.001), and dementia (43% vs. 29%, *p* = 0.026) were significantly more common in non-survivors. Prevalent delirium (20% vs. 4%, *p* < 0.001), hypokinetic delirium (20% vs. 5%, *p* < 0.001), and incident delirium (28% vs. 17%, *p* = 0.034) were markedly more frequent among patients who died. The number of daily medications was slightly lower in non-survivors (median 6 vs. 7, *p* = 0.027).

Sex-stratified comparisons between survivors and non-survivors (Table 3) showed that in males, non-survivors had higher CFS scores (*p* < 0.001) and more frequent severe frailty (45% vs. 13%, *p* < 0.001), prevalent delirium (28% vs. 4%, *p* < 0.001), hypokinetic delirium (17% vs. 3%, *p* = 0.002), cancer (59% vs. 29%, *p* = 0.002), anemia (45% vs. 23%, *p* = 0.011), and dementia (45% vs. 22%, *p* = 0.009). In females, non-survivors more often had severe frailty (34% vs. 18%, *p* = 0.024), higher CIRS-LIVER scores (*p* = 0.009), prevalent delirium (13% vs. 4%, *p* = 0.037), incident delirium (38% vs. 19%, *p* = 0.015), and hypokinetic delirium (22% vs. 6%, *p* = 0.001).

Among patients with delirium (n = 117), 73% had incident delirium and 27% prevalent delirium (Table 4). Those with prevalent delirium had higher CFS scores (median 7 vs. 6, *p* = 0.020) and experienced higher mortality (38% vs. 12%, *p* = 0.004). Sex-stratified analysis showed that mortality in males with prevalent delirium reached 50%, compared with 4% in those with incident delirium (*p* < 0.001), while in females the difference was not statistically significant (25% vs. 15%, *p* = 0.454).

In multivariable Cox regression (Table 5), independent predictors of in-hospital mortality included prevalent delirium (HR 4.66, 95% CI 2.42–8.96, *p* < 0.001), severe frailty (CFS ≥ 7) (HR 2.26, 95% CI 1.32–3.87, *p* = 0.003), CIRS-LIVER ≥ 2 (HR 2.05, 95% CI 1.18–3.56, *p* = 0.010), and cancer (HR 1.83, 95% CI 1.07–3.14, *p* = 0.026).

Sex-stratified Cox models (Table 6) revealed that in males, prevalent delirium (HR 10.23, 95% CI 4.25–24.59) and cancer (HR 2.49, 95% CI 1.18–5.25) were the most significant predictors. In females, hypokinetic delirium (HR 3.67, 95% CI 1.56–8.67) and CIRS-LIVER ≥ 2 (HR 2.75, 95% CI 1.30–5.79) were independently associated with death.

Multivariable logistic regression (Table 7) confirmed prevalent delirium (OR 5.25, 95% CI 2.27–12.18), CFS score (OR 1.35, 95% CI 1.11–1.65), CIRS-LIVER (OR 1.34, 95% CI 1.08–1.65), CIRS-SI ≥ 3 (OR 1.90, 95% CI 1.05–3.46), and cancer (OR 1.89, 95% CI 1.01–3.54) as independent predictors.

Sex-stratified logistic regression (Table 8) showed that in males, severe frailty (OR 4.39), prevalent delirium (OR 7.07), cancer (OR 3.43), and anemia (OR 3.16) were associated with death, while in females, CIRS-LIVER (OR 1.64), CIRS-SI ≥ 3 (OR 2.56), and hypokinetic delirium (OR 5.33) were the strongest predictors.

**Figure 1:**
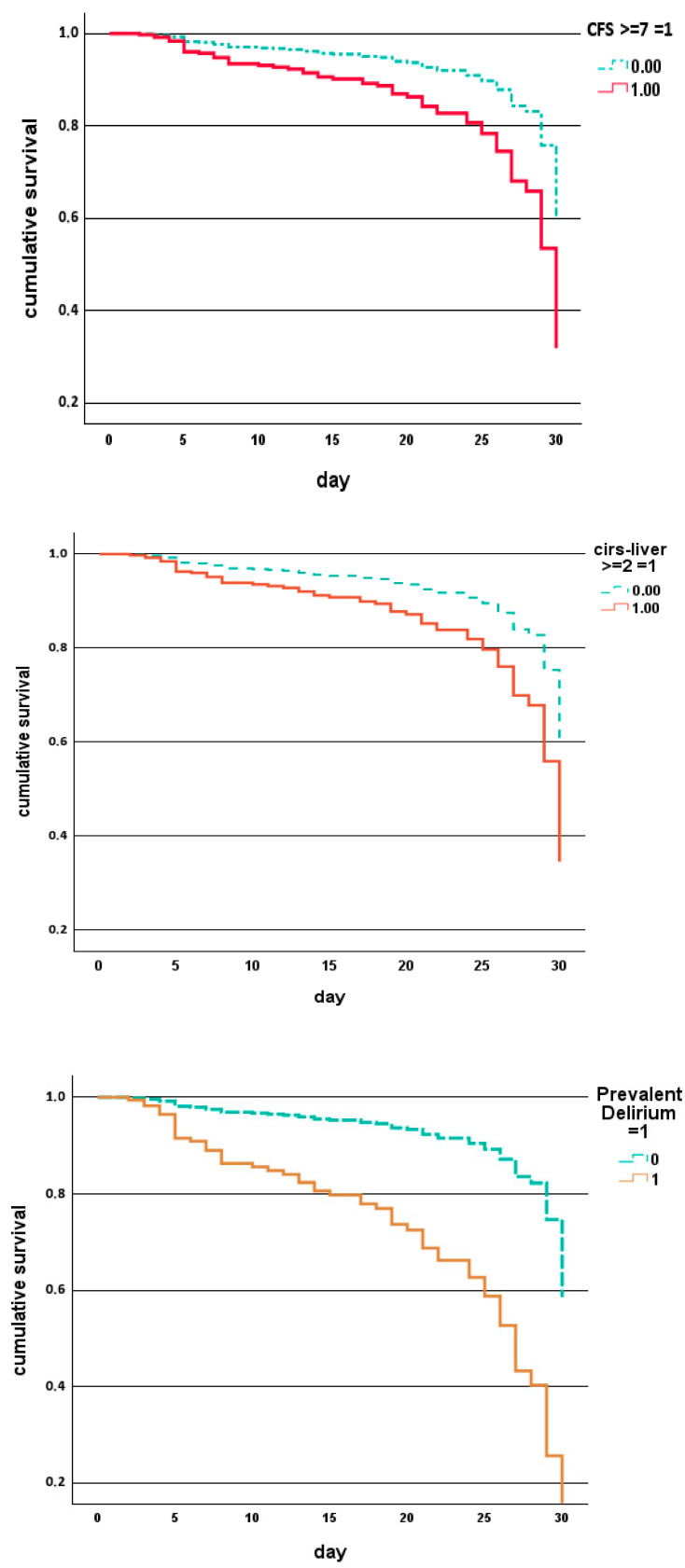

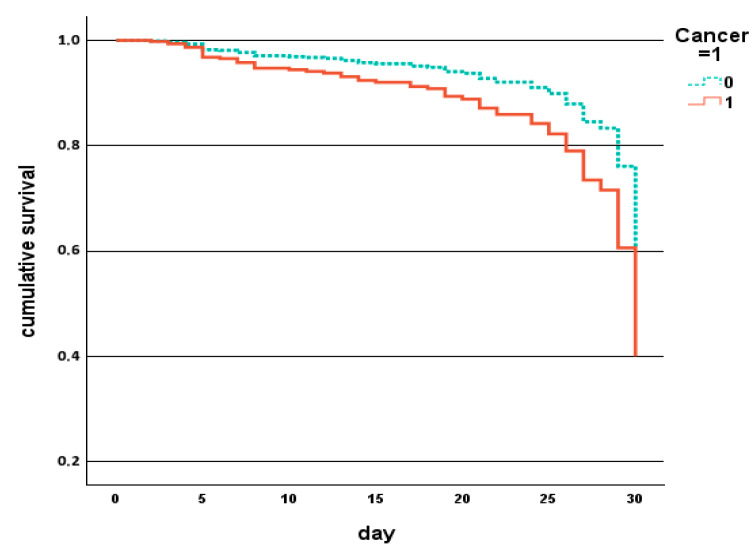
Cumulative survival (Cox regression) in patients with CFS ≥ 7 and <7, CIRS-LIVER ≥ 2 and <2, with and without cancer, and with and without prevalent delirium (parameters adjusted by the other parameters in Table 5).

**Figure 2:**
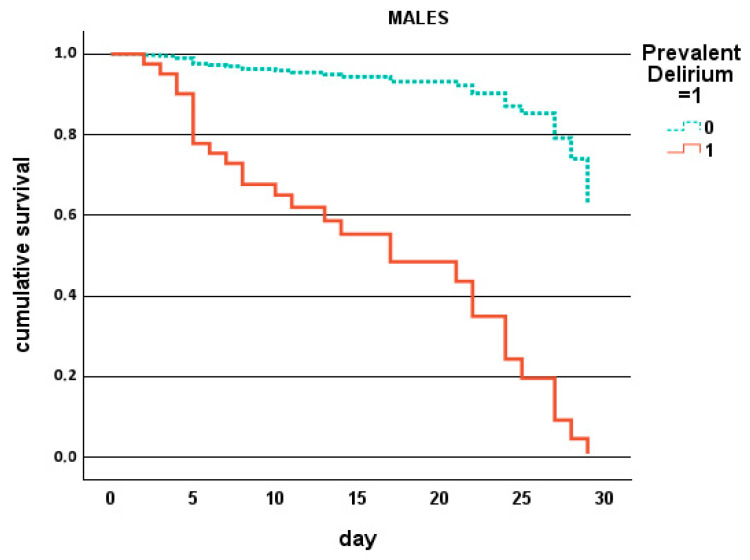

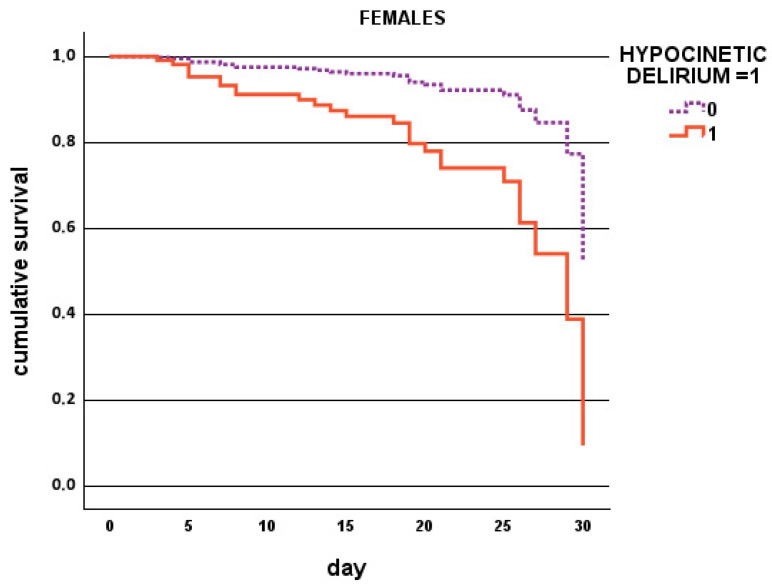
Cumulative survival (Cox regression) in male patients with/without prevalent delirium and in female patients with/without hypokinetic delirium (Table 6).

Cumulative survival (Cox regression), as shown in Figure 1, demonstrated significantly reduced survival for patients with severe frailty, prevalent delirium, chronic liver disease, or cancer, while Figure 2 shows that this can be observed in males and females with prevalent and hypokinetic delirium, respectively. Decision tree analysis identified subgroups at particularly high risk, with the highest mortality (37.5%) being observed among patients with prevalent delirium, followed by those with incident hypokinetic delirium (25%) and those with CIRS-LIVER ≥ 2 plus cancer (31.7%).

## 4. Discussion

In this prospective observational study of 556 older inpatients, we identified prevalent delirium, severe frailty, liver disease severity, and cancer as the strongest independent predictors of in-hospital mortality. Sex-stratified analyses revealed striking differences: in men, prevalent delirium and cancer dominated the risk profile, whereas in women, hypokinetic delirium and liver disease severity were the most influential factors. These findings confirm and extend previous evidence on the prognostic role of both delirium and frailty in acutely hospitalized older adults [13,14].

The overall prevalence of delirium (21% overall; 18% in men; and 23% in women) aligns with prior studies reporting rates between 14% and 38% in similar settings [15]. Notably, prevalent delirium was associated with a four- to tenfold increase in mortality risk, with the strongest effect being observed in men. This sex-specific impact may reflect differences in comorbidity profiles, baseline disease severity, or biological vulnerability, as men in our study exhibited a greater burden of cardiovascular, hepatic, and oncologic conditions. Our analysis also confirmed the prognostic weight of hypokinetic delirium, especially in women, where it conferred more than a fivefold increase in mortality risk. This subtype is known to be linked to greater illness severity, immobility, and complications such as aspiration pneumonia [16].

Frailty emerged as another key determinant of mortality, with severe frailty (CFS ≥ 7) doubling the risk of death. This is consistent with the well-established role of frailty as a marker of diminished physiological reserve and increased vulnerability to acute stressors [17,18,19,20]. Interestingly, while severe frailty was strongly predictive in men, in women, its prognostic weight was attenuated when hypokinetic delirium and liver disease severity were considered, suggesting different pathways to poor outcomes between sexes.

Liver disease severity, assessed through the CIRS-LIVER score, independently predicted mortality in the overall cohort and in women, but not in men. This could be related to a greater susceptibility of women to the systemic effects of liver dysfunction, possibly due to differences in body composition, inflammatory response, or nutritional status. By contrast, cancer emerged as a stronger predictor in men, suggesting sex-related interactions between frailty, comorbidities, and cancer-related outcomes.

We observed an association between a higher medication count and in-hospital mortality. Polypharmacy may exert direct adverse effects (drug–drug interactions, cumulative anticholinergic/sedative burden) and simultaneously reflect underlying disease complexity and care intensity. Because medication count can act as a proxy for illness severity, residual confounding is likely. Future work should test non-linear relationships, evaluate class-specific effects (e.g., psychoactive, anticholinergic, antithrombotic agents), and consider metrics of treatment complexity (e.g., medication appropriateness indices) to disentangle causality from confounding by indication.

The identification of distinct high-risk subgroups through decision tree analysis further emphasizes the clinical importance of integrating delirium subtype, frailty, and comorbidity patterns in prognostic assessment. Patients with prevalent delirium had the highest mortality risk, followed by those with incident hypokinetic delirium or a combination of severe liver disease and cancer. Such an approach can enhance early identification of high-risk patients and inform tailored interventions, ranging from closer monitoring to timely palliative care referral.

Our results underscore the need for systematic delirium screening at admission, close monitoring of high-risk subtypes, and the incorporation of frailty and comorbidity indices into routine prognostic evaluation. Future studies should investigate whether targeted interventions for these high-risk phenotypes can improve survival and functional outcomes. Additionally, the biological mechanisms underlying the observed sex-specific associations warrant further exploration, including hormonal influences, immune response differences, and disparities in healthcare utilization.

Our findings corroborate prior work linking CFS to short-term mortality and extend the evidence by distinguishing prevalent from incident delirium and quantifying the prognostic impact of hypoactive delirium, particularly in women. Unlike most internal medicine cohorts, we demonstrate an independent association between liver disease severity and in-hospital death after adjustment for frailty and comorbidities, suggesting an additive hepatic vulnerability.

This single-center design and 7-month enrollment window may limit the external validity. Case mix, care pathways, and resource availability can differ across hospitals and seasons; therefore, effect sizes, particularly for delirium subtypes and liver disease severity, should be interpreted with caution and validated in multicenter cohorts. Nevertheless, consecutive inclusion of all eligible patients reduces selection bias and reflects real-world internal medicine practice. We only assessed in-hospital mortality; the lack of functional trajectories, 30-/90-day readmissions, and long-term survival limits clinical applicability. Future work should link frailty and delirium subtypes to post-discharge outcomes.

Key potential confounders, such as nutritional status, depressive symptoms, and care dependency, were unavailable, introducing residual confounding despite adjustments for comorbidity burden and frailty

The sex-specific results may reflect distinct comorbidity patterns (e.g., higher cancer burden in men; greater impact of liver disease in women), phenotype-dependent delirium risks (hypoactive subtype, immobility, aspiration), and biological differences in inflammatory response and physiological reserve. Social factors, including care dependency and recognition/treatment patterns of hypoactive delirium, may further contribute. These hypotheses warrant mechanistic and health-service-related research.

Our sex-stratified analyses should be interpreted as exploratory. While differences in comorbidity patterns (e.g., cancer burden in men, the impact of liver disease severity in women) and phenotype-dependent delirium risks (notably hypoactive presentations) are plausible contributors, we lacked direct measures of sex-specific biological pathways (e.g., hormones, immune profiles). Consequently, the observed differences warrant confirmation in adequately powered cohorts with prespecified sex analyses rather than causal interpretation.

These findings highlight the relevance of a precision medicine approach, where individualized prognostic assessment that integrates frailty, delirium subtypes, and comorbidity burden may optimize clinical decision-making and improve outcomes in older hospitalized patients.

## 5. Conclusions

In this prospective cohort of older adults who were admitted to an internal medicine ward, frailty and delirium remained key correlates of in-hospital mortality. Sex-related patterns and the association between medication count and mortality are hypothesis-generating and require validation in prespecified analyses. Our findings should inform risk stratification while encouraging multicenter studies with standardized outcomes and comprehensive adjustment for confounding factors.

## Figures and Tables

**Table 1:** General characteristics of patients aged ≥ 70 and admitted to internal medicine ward and their comparison after stratification by gender.

Parameter	Overall Population N. 556	Males N. 231 (42%)	Females N. 325 (58%)	*p*
Age, years	85 (80–89)	83 (78–88)	86 (80–90)	**0.001**
Chronic illnesses, number	5 (4–7)	5 (4–7)	5 (3–7)	0.195
Drugs, number	6 (5–9)	6 (4–8)	6 (5–9)	0.696
CFS	5 (4–6)	5 (4–6)	5 (4–6)	0.285
CFS ≥ 7, %	19	17	19	0.536
Chronic illnesses				
Hypertension, %	70	66	73	0.106
Cardiomyopathy, %	48	49	46	0.502
Arrythmia, %	37	39	35	0.266
Anemia, %	26	26	26	0.871
COPD, %	30	36	26	**0.010**
Diabetes, %	28	35	22	**<0.001**
Obesity, %	7	9	6	0.201
Dyslipidemia, %	21	24	18	0.066
Parkinsonism, %	9	9	9	0.816
Previous stroke, %	15	13	16	0.251
CKD, %	16	20	13	**0.025**
Cancer, %	26	33	21	**0.001**
Dementia, %	30	26	34	**0.036**
Psychiatric disease, %	18	15	20	0.168
Peripheral artery disease, %	30	35	26	**0.024**
Liver disease, %	13	16	10	**0.015**
Gastrointestinal disease, %	25	27	24	0.383
Osteoporosis, %	10	3	15	**<0.001**
Arthrosis, %	25	16	32	**<0.001**
Admission diagnosis				
Pneumonia, %	13	13	13	0.781
Infections (urinary, sepsis, other acute infections), %	9	10	8	0.529
Heart failure, %	14	13	15	0.728
Syncope, tachyarrhythmia, coronary syndrome, stroke, %	7	7	7	0.899
COPD, %	4	3	5	0.166
CKD, %	2	1	2	0.615
Cirrhosis, pancreatitis, gastrointestinal bleeding, cholecystitis, diverticulitis, %	8	8	7	0.858
DVT or lower limb ischemia, %	1	0	2	0.340
Falls with or without fractures, %	6	4	7	0.146
Overall decay, %	1	2	0	0.107
Other, %	36	38	34	0.439
Delirium, %	21	18	23	0.108
Prevalent delirium, %	6	7	5	0.318
Delirium incident, %	18	14	21	**0.032**
Hyperkinetic delirium, %	10	10	10	0.995
Mixed delirium	5	3	6	0.092
Hypokinetic delirium, %	7	5	8	0.245
Length of hospital stay, day	15 (8–23)	15 (7–22)	16 (8–23)	0.264
Death, %	11	13	10	0.305

Abbreviations: CFS, Clinical Frailty Scale; COPD, chronic obstructive pulmonary disease; DVT, deep vein thrombosis; CKD, chronic kidney disease. Data are expressed as median and IQR or percentage. *p* values are calculated with Mann–Whitney test for continuous variables and chi-square test or Fisher’s exact test for dichotomous variables. *p* < 0.05 is indicated in bold.

**Table 2:** Comparison of the characteristics of the population aged ≥ 70 and admitted to internal medicine ward, stratified by survivors and non-survivors.

	Survivors N. 495 (89%)	Non-Survivors N. 61 (11%)	*p*
Age, years	85 (79–89)	86 (80–89)	0.303
Females, %	59	52	0.306
Chronic illnesses, number	5 (3–7)	5 (4–7)	0.708
Drugs, number	7 (5–9)	6 (4–8)	**0.027**
CFS	5 (4–6)	6 (5–7)	**<0.001**
CFS ≥ 7, %	16	39	**<0.001**
CIRS-SI ≥ 3, %	31	55	**<0.001**
Chronic illnesses			
Hypertension, %	71	59	**0.049**
Cardiomyopathy, %	49	41	0.263
Arrythmia, %	36	41	0.469
Anemia, %	25	36	0.056
COPD, %	30	31	0.900
Diabetes, %	28	21	0.260
Obesity, %	7	5	0.495
Dyslipidemia, %	21	13	0.129
Parkinsonism, %	9	8	0.854
Previous stroke, %	14	20	0.237
CKD, %	16	16	0.968
Cancer, %	24	44	**<0.001**
Dementia, %	29	43	**0.026**
Psychiatric disease, %	18	13	0.308
Peripheral artery disease, %	30	33	0.604
Liver disease, %	11	23	**0.008**
Gastrointestinal disease, %	25	26	0.876
Osteoporosis, %	11	7	0.332
Arthrosis, %	26	20	0.276
Admission diagnosis			
Pneumonia, %	13	11	0.745
Infections (urinary, sepsis, other acute infections), %	9	10	0.727
Heart failure, %	15	7	0.074
Syncope, tachyarrhythmia, coronary syndrome, stroke, %	7	10	0.427
COPD, %	4	2	0.495
CKD, %	1	3	0.259
Cirrhosis, pancreatitis, gastrointestinal bleeding, cholecystitis, diverticulitis, %	8	7	1.000
DVT or lower limb ischemia, %	1	2	1.000
Falls with or without fractures, %	6	0	**0.038**
Overall decay, %	1	3	0.174
Other, %	35	46	0.083
Delirium, %	19	36	**0.002**
Prevalent delirium, %	4	20	**<0.001**
Delirium incident, %	17	28	**0.034**
Hyperkinetic delirium, %	9	8	0.780
Hypokinetic delirium, %	5	20	**<0.001**
Mixed delirium, %	4	8	0.200
Length of hospital stay, day	15 (8–23)	15 (7–25)	0.739

Abbreviations: CFS, Clinical Frailty Scale; CIRS-SI = Cumulative Illness Rating Scale Severity Index; COPD = Chronic Obstructive Pulmonary Disease; CKD = Chronic Kidney Disease. Data are expressed as median and IQR or percentage. *p* values are calculated with Mann–Whitney test for continuous variables and chi-square test or Fisher’s exact test for dichotomous variables. *p* < 0.05 is indicated in bold.

**Table 3:** Gender stratification of Table 2 parameters that are significantly different between survivors and non-survivors.

	Males N. 230		Females N. 325			
	Survivors N. 201 (87%)	Non-Survivors N. 29 (13%)	*p*	Survivors N. 293 (90%)	Non-Survivors N. 32 (10%)	*p*
Age, years	83 (78–88)	85 (81–89)	0.081	86 (80–90)	86 (80–90)	0.869
Chronic illnesses, number	5 (4–7)	5 (4–7)	0.667	5 (3–7)	5 (4–7)	0.847
Drugs, number	7 (5–9)	5 (4–6)	**0.020**	7 (5–9)	6 (4–8)	**0.365**
CFS	5 (4–6)	6 (5–7)	**<0.001**	5 (4–6)	6 (4–7)	**0.026**
CFS ≥ 7, %	13	45	**<0.001**	18	34	**0.024**
Anemia, %	23	45	**0.011**	26	28	0.790
Cancer, %	29	59	**0.002**	20	31	0.145
Dementia, %	22	45	**0.009**	33	41	0.395
Liver disease, %	16	21	0.520	8	25	**0.002**
CIRS-LIVER ≥ 2, %	21	38	**0.036**	11	35	**<0.001**
Delirium, %	16	31	**0.047**	22	41	**0.015**
Prevalent delirium, %	4	28	**<0.001**	4	13	**0.037**
Delirium incident, %	13	17	0.582	19	38	**0.015**
Hypokinetic delirium, %	3	17	**0.002**	6	22	**0.001**

Abbreviations: CFS, Clinical Frailty Scale; CIRS-LIVER = Cumulative Illness Rating Scale—liver. Data are expressed as median and IQR or percentage. *p* values are calculated with Mann–Whitney test for continuous variables and chi-square test or Fisher’s exact test for dichotomous variables. *p* < 0.05 is indicated in bold.

**Table 4:** Patients with delirium (N. 117), stratified by incident and prevalent delirium.

	Delirium Incident N. 85 (73%)	Prevalent Delirium N. 32 (27%)	*p*
Age, years	88 (84–92)	84 (79–89)	**0.037**
Females, %	71	50	0.062
CFS	6 (5–7)	7 (6–7)	**0.020**
CFS ≥ 7, %	29	50	0.062
Hyperkinetic delirium, %	51	31	0.096
Mixed delirium, %	21	28	0.583
Hypokinetic delirium, %	28	41	0.288
Death, %	12	38	**0.004**
Death in males, %	4	50	**<0.001**
Death in females, %	15	25	0.454
Death in patients without hypokinetic delirium, %	7	32	**0.010**
Death in patients with hypokinetic delirium, %	25	46	0.200

Abbreviations: CFS = Clinical Frailty Scale. Data are expressed as median and IQR or percentage. *p* values are calculated with Mann–Whitney test for continuous variables and Yates’ chi-square test or Fisher’s exact test for dichotomous variables. *p* < 0.05 is indicated in bold.

**Table 5:** Risk of death in hospital in geriatric patients, tested with Cox regression multivariate analysis, using stepwise method.

	*p*	Hazard Ratio	95% CI for Hazard Ratio
CFS ≥ 7, %	0.003	2.258	1.319–3.868
Prevalent delirium, %	<0.001	4.660	2.424–8.957
CIRS-LIVER ≥ 2, %	0.010	2.052	1.184–3.555
Cancer, %	0.026	1.835	1.073–3.136

Abbreviations: CFS = Clinical Frailty Scale; CIRS-LIVER = Cumulative Illness Rating Scale—liver.

**Table 6:** Risk of death in hospital in geriatric patients, stratified for sex and tested with Cox regression multivariate analysis, using stepwise method.

	*p*	Hazard Ratio	95% CI for Hazard Ratio
Males			
Prevalent delirium, %	<0.001	10.227	4.253–24.588
Cancer, %	0.017	2.486	1.176–5.254
Females			
Hypokinetic delirium, %	0.003	3.672	1.556–8.668
CIRS-LIVER ≥ 2, %	0.008	2.745	1.302–5.786

Abbreviations: CIRS-LIVER = Cumulative Illness Rating Scale—liver.

**Table 7:** Factors that are independently associated with mortality in hospitalized patients, tested with Logistic Regression Model Multivariate Analysis, using stepwise method.

	*p*	Odds Ratio	95% CI for Odds Ratio
CFS	0.003	1.350	1.107–1.646
Prevalent delirium, %	<0.001	5.254	2.267–12.177
CIRS-LIVER	0.007	1.337	1.082–1.652
CIRS-SI ≥ 3, %	0.034	1.904	1.049–3.458
Cancer, %	0.048	1.886	1.006–3.536

Abbreviations CFS = Clinical Frailty Scale; CIRS-LIVER = Cumulative Illness Rating Scale—liver; CIRS = Cumulative Illness Rating Scale; -SI = Severity Index.

**Table 8:** Factors that are independently associated with mortality in hospitalized patients, stratified for sex and tested with Logistic Regression Model Multivariate Analysis, using stepwise method.

	*p*	Odds Ratio	95% CI for Odds Ratio
Males			
CFS ≥ 7, %	0.003	4.393	1.655–11.663
Prevalent delirium, %	0.003	7.073	1.968–25.421
Cancer, %	0.007	3.435	1.404–8.403
Anemia, %	0.015	3.162	1.255–7.966
Females			
CIRS-LIVER	<0.001	1.640	1.231–2.185
CIRS-SI ≥ 3, %	0.021	2.558	1.155–5.667
Hypokinetic delirium, %	0.001	5.328	1.921–14.777

Abbreviations: CIRS-LIVER = Cumulative Illness Rating Scale—liver; CFS = Clinical Frailty Scale; CIRS = Cumulative Illness Rating Scale; -SI = Severity Index.

## Data Availability

The research data associated with this paper is available locally at the site and accessible if granted approval.

## References

[1] Gutiérrez-Valencia M., Izquierdo M., Cesari M., Casas-Herrero Á., Inzitari M., Martínez-Velilla N. (2018). The relationship between frailty and polypharmacy in older people: A systematic review. Br. J. Clin. Pharmacol..

[2] Zhao Y., Wang J., Zhu X., Zhang X., Zhang Y., Zhang W., Dong Y. (2023). Multimorbidity and polypharmacy in hospitalized older patients: A cross-sectional study. BMC Geriatr..

[3] McDonagh J., Lindley R.I., Byth K., John R., Ferguson C. (2025). Frailty in older adults admitted to hospital: Outcomes from the Western Sydney Clinical Frailty Registry. BMC Geriatr..

[4] Wen Y.C., Chen L.K., Hsiao F.Y. (2017). Predicting mortality and hospitalization of older adults by the multimorbidity frailty index. PLoS ONE..

[5] Marcantonio E.R. (2017). Delirium in Hospitalized Older Adults. N. Engl. J. Med..

[6] O’Keeffe S., Lavan J. (1997). The prognostic significance of delirium in older hospital patients. J. Am. Geriatr. Soc..

[7] Pérez-Ros P., Plaza-Ortega N., Martínez-Arnau F.M. (2025). Mortality Risk Following Delirium in Older Inpatients: A Systematic Review and Meta-Analysis. Worldviews Evid. Based Nurs..

[8] Connell J.M., Duggan M.C., Wilson J.E. (2023). Why We Must Prevent and Appropriately Manage Delirium. AMA J. Ethics.

[9] Rockwood K., Song X., MacKnight C., Bergman H., Hogan D.B., McDowell I., Mitnitski A. (2005). A global clinical measure of fitness and frailty in elderly people. CMAJ.

[10] Bellelli G., Morandi A., Davis D.H., Mazzola P., Turco R., Gentile S., Ryan T., Cash H., Guerini F., Torpilliesi T. (2014). Validation of the 4AT, a new instrument for rapid delirium screening: A study in 234 hospitalised older people. Age Ageing.

[11] Meagher D. (2009). Motor subtypes of delirium: Past, present and future. Int. Rev. Psychiatry.

[12] Pilotto A., Cella A., Pilotto A., Daragjati J., Veronese N., Musacchio C., Mello A.M., Logroscino G., Padovani A., Prete C. (2017). Three Decades of Comprehensive Geriatric Assessment: Evidence Coming From Different Healthcare Settings and Specific Clinical Conditions. J. Am. Med. Dir. Assoc..

[13] Yan E., Veitch M., Saripella A., Alhamdah Y., Butris N., Tang-Wai D.F., Tartaglia M.C., Nagappa M., Englesakis M., He D. (2023). Association between postoperative delirium and adverse outcomes in older surgical patients: A systematic review and meta-analysis. J. Clin. Anesth..

[14] Aung Thein M.Z., Pereira J.V., Nitchingham A., Caplan G.A. (2020). A call to action for delirium research: Meta-analysis and regression of delirium associated mortality. BMC Geriatr..

[15] Schreiber N., Eichlseder M., Orlob S., Klivinyi C., Zoidl P., Pichler A., Eichinger M., Fandler-Höfler S., Scholz L., Baumgartner J. (2024). Sex specific differences in short-term mortality after ICU-delirium. Crit. Care.

[16] Cavallazzi R., Saad M., Marik P.E. (2012). Delirium in the ICU: An overview. Ann. Intensive Care.

[17] Lu Y.W., Chang C.C., Chou R.H., Lee W.J., Chen L.K., Huang P.H., Lin S.J. (2024). Sex differences in the frailty phenotype and mortality in the I-Lan longitudinal aging study cohort. BMC Geriatr..

[18] Penfold R.S., Squires C., Angus A., Shenkin S.D., Ibitoye T., Tieges Z., Neufeld K.J., Avelino-Silva T.J., Davis D., Anand A. (2024). Delirium detection tools show varying completion rates and positive score rates when used at scale in routine practice in general hospital settings: A systematic review. J. Am. Geriatr. Soc..

[19] Javadzadeh D., Karlson B.W., Alfredsson J., Ekerstad E., Hellberg J., Herlitz J., Ekerstad N. (2024). Clinical Frailty Scale score is a predictor of short-, mid- and long-term mortality in critically ill older adults (≥70 years) admitted to the emergency department: An observational study. BMC Geriatr..

[20] Pilotto A., Aprile P.L., Veronese N., Lacorte E., Morganti W., Custodero C., Piscopo P., Fabrizi E., Gatta F.D., Merlo A. (2024). The Italian guideline on comprehensive geriatric assessment (CGA) for the older persons: A collaborative work of 25 Italian Scientific Societies and the National Institute of Health. Aging Clin. Exp. Res..

